# Vascular disruption and blood–brain barrier dysfunction in intracerebral hemorrhage

**DOI:** 10.1186/2045-8118-11-18

**Published:** 2014-08-10

**Authors:** Richard F Keep, Ningna Zhou, Jianming Xiang, Anuska V Andjelkovic, Ya Hua, Guohua Xi

**Affiliations:** 1 Department of Neurosurgery, University of Michigan, Ann Arbor, Michigan 48109-2200, USA; 2 Department of Molecular & Integrative Physiology, University of Michigan, Ann Arbor, USA; 3 Department of Pharmacology, Yunnan University of Traditional Chinese Medicine, Kunming, China; 4 Department of Pathology, University of Michigan, Ann Arbor, USA

**Keywords:** Intracerebral hemorrhage, Hematoma expansion, Blood–brain barrier, Endothelium, Tight junction, Thrombin, Hemoglobin, Iron

## Abstract

This article reviews current knowledge of the mechanisms underlying the initial hemorrhage and secondary blood–brain barrier (BBB) dysfunction in primary spontaneous intracerebral hemorrhage (ICH) in adults. Multiple etiologies are associated with ICH, for example, hypertension, Alzheimer’s disease, vascular malformations and coagulopathies (genetic or drug-induced). After the initial bleed, there can be continued bleeding over the first 24 hours, so-called hematoma expansion, which is associated with adverse outcomes. A number of clinical trials are focused on trying to limit such expansion. Significant progress has been made on the causes of BBB dysfunction after ICH at the molecular and cell signaling level. Blood components (e.g. thrombin, hemoglobin, iron) and the inflammatory response to those components play a large role in ICH-induced BBB dysfunction. There are current clinical trials of minimally invasive hematoma removal and iron chelation which may limit such dysfunction. Understanding the mechanisms underlying the initial hemorrhage and secondary BBB dysfunction in ICH is vital for developing methods to prevent and treat this devastating form of stroke.

## Introduction

Each year, there are approximately 800,000 strokes in the USA alone. Although ischemic stroke is the predominant form, approximately 10-15% of these strokes are intracerebral hemorrhage (ICH) and ICH accounts for an even greater percentage of strokes in Asians [1]. ICH is associated with a higher morbidity and mortality compared to ischemic stroke [1]. The occurrence of ICH is also a major concern in the prevention and treatment of ischemia. Thus, anticoagulant treatment for prevention of ischemia (brain and heart) is a growing cause of ICH [2] and the occurrence of symptomatic ICH is a major concern in using tissue plasminogen activator for treating ischemic stroke [3].

Although there are a number of ongoing clinical trials [4], there is currently no FDA approved treatment for ICH. Thus, understanding the mechanisms of brain injury after ICH is important. Vascular disruption is the initial cause of ICH and blood–brain barrier (BBB) dysfunction (with associated edema and leukocyte extravasation) is a secondary consequence [5]. It is vital to understand the mechanisms underlying these two events in ICH.

The aim of this article is to review current knowledge about the mechanisms that may underlie the initial hemorrhage and the secondary BBB dysfunction in adult primary spontaneous ICH. With regards to the latter, there is evidence that cerebral ischemia is not an important component of brain injury after ICH in animal models and in man [5]. Thus, while there is some overlap, there are distinct differences in the mechanisms underlying BBB dysfunction between ischemic stroke and ICH.

During this review, the term BBB dysfunction is used to describe changes in BBB function post-ICH because some of those changes can have deleterious effects (e.g. brain edema may result in brain herniation and death). However, it should be noted that the changes in function may also be adaptive (e.g. infiltrating macrophages may be involved in hematoma resolution). Whether the BBB effects are detrimental or beneficial may depend on hematoma size [6], being beneficial for microbleeds but having adverse effects with large bleeds.

### ICH Etiology and hematoma expansion

Hypertension is the most common cause of spontaneous ICH, accounting for about two thirds of hemorrhages. ICH is also linked to amyloid angiopathy, brain tumors and various vascular malformations (e.g. aneurysms, ateriovenous malformations, cerebral cavernous malformations) [7] (Table 1). Increasingly, anticoagulants are also a cause. Their use now accounts for almost 20% of ICH cases in the USA [2]. Location has important impact on the frequency and outcome of ICH. Most ICH is ganglionic or lobar [7] and hindbrain hemorrhages have a worse outcome.

**Table 1:** Major causes of intracerebral hemorrhage

**Etiology**	**Proposed mechanism underlying hemorrhage**
Hypertension	Vascular remodeling, formation microaneurysms.
Amyloid angiopathy	β-amyloid induced vascular damage.
Ateriovenous malformations	Weakened vascular wall.
Cerebral cavernous malformations	Weakened vascular wall.
Anti-coagulant usage	Loss of coagulation in response to bleeding (microbleeds).
Ischemic stroke*	Ischemia/reperfusion induced vascular injury
Traumatic brain injury*	Physical disruption of vessels
Tumors*	Abnormal vasculature

*In addition to primary spontaneous ICH, a hemorrhage can also be secondary to other adverse events including an ischemic stroke (hemorrhagic transformation), brain tumors and traumatic brain injury. Such hemorrhages can have an important role in brain injury following such events. In ischemic stroke, the risk of a symptomatic ICH is enhanced by inducing reperfusion with tissue plasminogen activator (tPA).

While most bleeding occurs at ictus, in about 30% of ICH patients there is further bleeding (hematoma expansion) during the course of the first day as shown by brain imaging [7,8]. Such hematoma expansion is associated with poor prognosis and it may, therefore, represent a therapeutic target [8].

### Secondary BBB dysfunction

Brain injury and death may result from the initial hemorrhage because of increased intracranial pressure, brain herniation and physical disruption to the brain. However, as in ischemic stroke, ICH also induces secondary brain damage [5]. This includes BBB dysfunction as shown by evidence in animals and humans.

**Figure 1:**
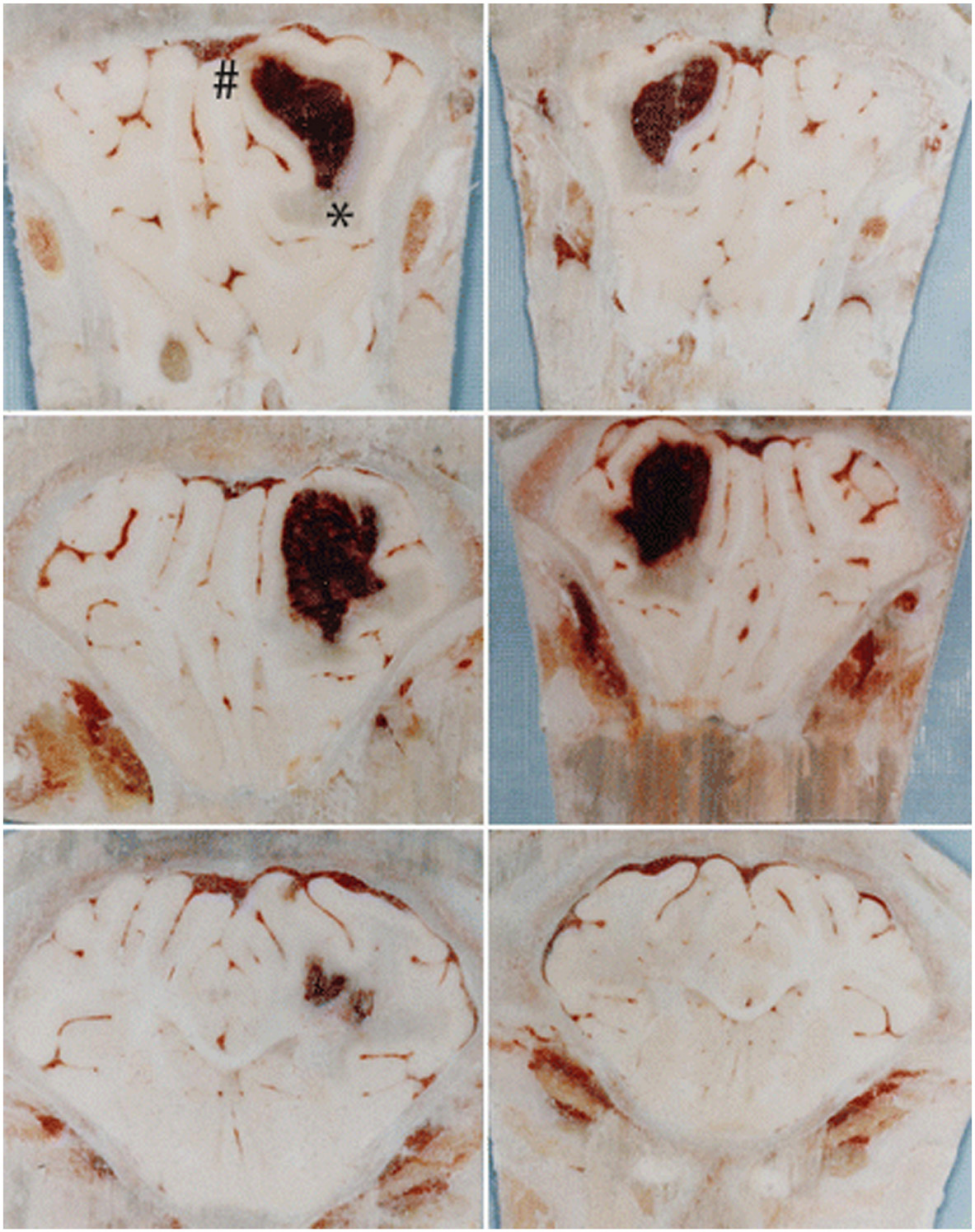
Photomicrographs showing both sides of serial coronal sections (thickness approximately 5 mm) containing hematoma (#) and edematous white matter (*) 24 hours after blood infusion in a pig brain frozen in situ. Perihematomal edema is present as blue-staining translucent regions in white matter adjacent to the hematoma. Evans blue staining is observed throughout the ipsilateral white matter and is indicative of increased BBB permeability and vasogenic edema development. Figure/legend reprinted with permission from *Journal of Neurosurgery*[11].

In animals, ICH has mostly been modeled by injecting blood directly into the brain or by intracerebral injection of collagenase. The latter causes bleeding (vascular disruption) via basement membrane degradation. After intracerebral injection of blood into brain, there is delayed BBB hyperpermeability (Figure 1). In rat, Yang *et al*. found no disruption at 4 hours but progressive disruption from 12 to 48 hours [9]. In pig, Wagner *et al*. found no disruption from 1 to 8 hours, but found disruption by 24 hours [10,11]. Following collagenase injection, there was vascular disruption (bleeding) by 30 minutes, but a prolonged lower level BBB hyperpermeability from 5 hours to 7 days [12]. In these animal models, hyperpermeability is associated with brain edema formation and an influx of inflammatory cells into the brain.

**Figure 2:**
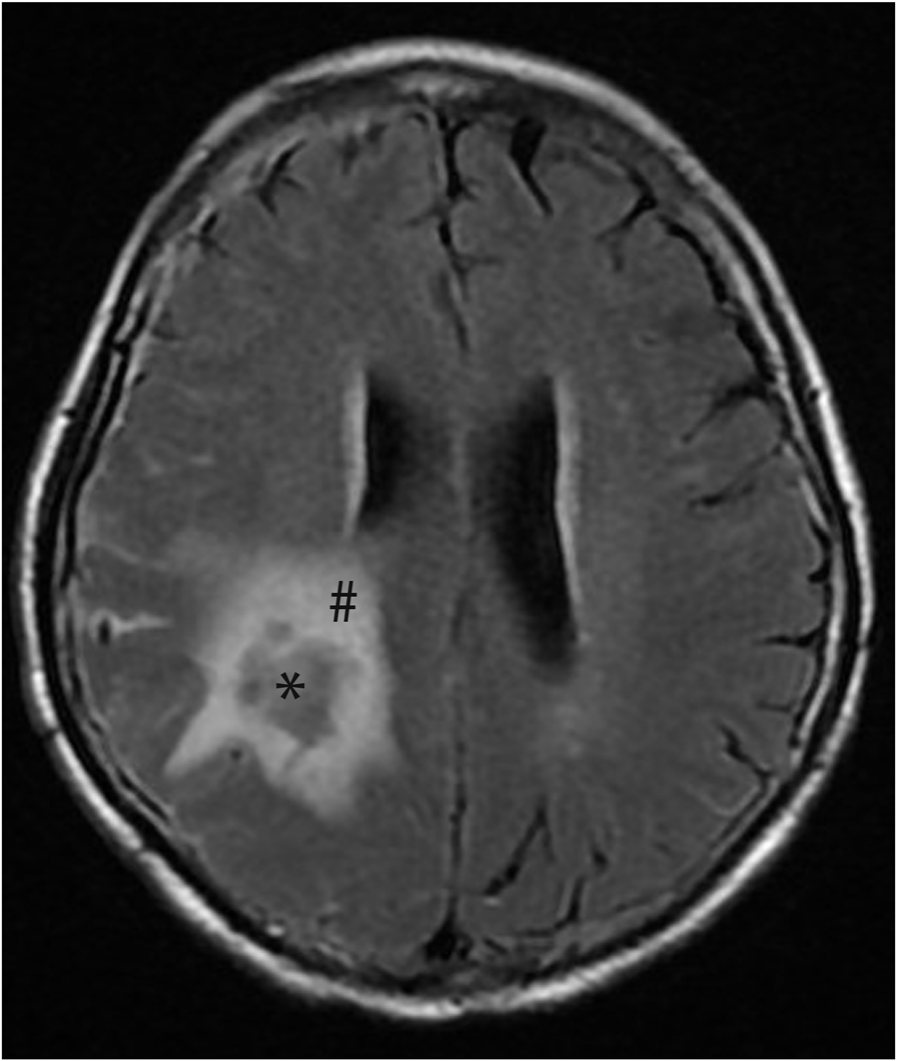
**Flair magnetic resonance imaging in a patient at day 1 after ICH.** Note the marked edema (^#^) surrounding the hematoma (*).

In human ICH, both acute and delayed vascular disruption occurs. As noted above, in some patients there is continued bleeding and the extravasation of contrast agent during the first 24 hours after ictus [8,13], but there is also more delayed disruption [14,15] which may be similar to that found after blood injection in animal models. As in animal models, human BBB disruption after ICH is associated with edema formation (Figure 2) and an influx of leukocytes into brain [5,7].

It is difficult to assess the importance of secondary BBB disruption in ICH. It will contribute to brain edema formation (vasogenic edema), it may participate in neuroinflammation by facilitating leukocyte migration into brain, and it will allow the entry of potentially harmful molecules into brain (e.g. prothrombin which is converted to thrombin and may participate in brain injury; see below). However, while there are experimental treatments that reduce BBB disruption and ameliorate ICH-induced brain injury, those treatments are not ‘BBB-specific’ and it may be that the beneficial effects come from parenchymal cells.

### Mechanisms underlying initial hemorrhage

#### Hypertension

In hypertension, the cause of ICH is thought to relate to remodeling of the cerebral vasculature. Thus, hypertension can cause microaneurysms at the bifurcation of arterioles and chronic elevation of intraluminal arterial pressure can cause small vessel wall damage [16,17]. Interestingly, in an animal model of ICH, salt fed stroke-prone spontaneously hypertensive rats, BBB hyperpermeability precedes hemorrhaging [18] suggesting there is a gradual weakening of vascular integrity that eventually leads to rupture. There is a genetic component to ICH [19]. Jeanne *et al*. [20] identified rare mutations in collagen type IV, a component of cerebrovascular basement membranes, that were associated with ICH. Recently, Woo *et al*. [21], identified 1q22 as a susceptibility locus for non-lobar ICH, although the precise gene involved is still uncertain. Lobar ICH is often due to cerebral amyloid angiopathy and that is discussed below.

#### Aneurysms

Cerebral aneurysms are balloon-like structures caused by a weakness in a blood vessel wall. It has been estimated that they are present in 3.6-6% of the population over 30 years old [22]. They are prone to rupture. Because of location, bleeding is usually into the subarachnoid space (causing subarachnoid hemorrhage; SAH), but ICH can occur [23]. The risk of bleeding is low in previously unruptured aneurysms, but aneurysm rupture accounts for about 27,000 SAH cases per year in the USA [23].

#### Cavernous malformations

**Figure 3:**
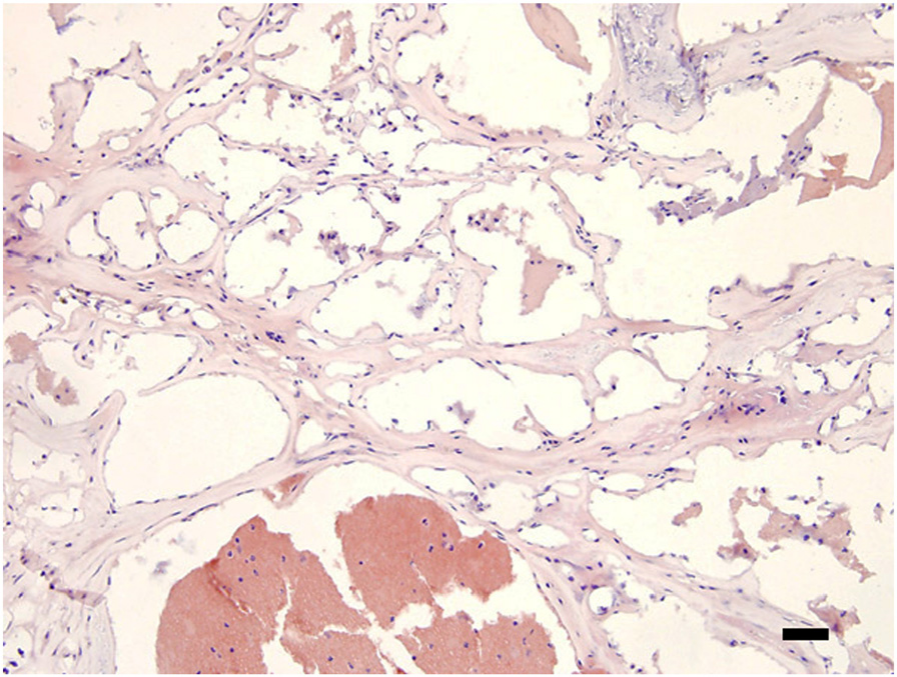
**Hematoxylin & eosin staining of a section from the brain of a patient with cerebral cavernous malformation (type CCM3).** Note the multiple blood vessels with little or no intervening parenchymal tissue. Scale bar = 200 μm.

Cerebral cavernous malformations (CCMs) are mulberry-shaped vascular lesions that occur in the capillary-venous vascular bed [24]. They form vascular channels with little or no intervening parenchymal tissue (Figure 3). The growth (size and number) and bleeding of these lesions can result in neurological symptoms. CCMs are generally sporadic but some are familial (~20%) and, in the latter, loss-of-function mutations have been identified in three genes: CCM1 (KRIT1), CCM2 (malcavernin, OSM, MGC4607) and CCM3 (PDCD10) [24,25]. CCM1, 2 and 3 can form a complex (the CCM complex signaling platform [25]) and regulate adhesion junction stability [24-26]. The loss of this regulation through mutations may be involved in both lesion growth and bleeding. It should be noted, however, that there are differences in the cellular distribution of CCM1, 2 and 3 [24,25] and they have important actions outside the CCM complex [24,25]. Also, there is evidence that CCMs regulate endothelial tight junction function either directly or indirectly via altered adhesion junction function [24]. While there is still much to be learnt about the function of CCM proteins, they do have important effects on endothelial junction proteins, and the loss of those effects that may lead to bleeding.

#### Ateriovenous malformations

Ateriovenous malformations (AVMs) are a tangle of blood vessels lacking a true capillary bed. These are prone to rupture and cerebral hemorrhage is the most severe AVM complication. A number of studies have examined genetic risk factors for hemorrhage. Polymorphisms in the *EPHB4* gene encoding a tyrosine kinase receptor involved in arterial-venous determination are associated with bleeding in AVMs [27]. In addition, polymorphisms in the *IL-6*, *TNFα* , *APOE* (the *APOEϵ2* allele) and *IL-1β* genes or gene promoters are associated with hemorrhage in AVMs [28]. As described below, inflammation impacts cerebrovascular integrity.

#### Amyloid angiopathy

Cerebral amyloid angiopathy (CAA) is the deposition of β-amyloid in small arteries, arterioles and capillaries of the cerebral cortex and leptomeninges [29-31]. β-amyloid is thought to have damaging effects on the vasculature resulting in microbleeds and symptomatic ICH, particularly lobar ICH [31,32]. CAA occurs in almost all Alzheimer’s disease patients, with severe CAA occurring in about 25% of patients [31]. Patients with the *APOEϵ2* or *ϵ4* alleles are at greater risk of ICH [31]. Polymorphism in the *CR1* gene, encoding the complement component (3b/4b) receptor 1, is associated with development of Alzheimer’s disease, likely because CR1 is involved in β-amyloid clearance [33]. Recently, Biffi *et al*. found that a variant in the CR1 gene was associated with increased risk of CAA-related ICH [34].

#### Infection

A recent study raises the interesting possibility that certain types of infection may also cause ICH. Nakano *et al*. [35] found that infection with a strain of *Streptococcus mutans* (serotype k) expressing a collagen binding protein (CBP) aggravated cerebral hemorrhage in mice and that the incidence of *S. mutans* expressing the CBP was higher in hemorrhagic stroke patients than control subjects. Nakano *et al*. found that CBP-expressing *S. mutans* accumulated in damaged vessels and that they inhibit collagen-induced platelet activation. The impact of infections with this strain of *S. mutans* in patients who are susceptible to ICH (e.g. people with CCMs and AVMs) deserves investigation.

#### Anticoagulants

A growing proportion of ICH cases are linked to anticoagulant use, particularly the vitamin K antagonist warfarin [1,2]. It has been estimated that there are about 2 million asymptomatic microhemorrhages a year in the USA [36] and the presence of an anticoagulant may transform a microbleed into a symptomatic ICH. The presence of microbleeds increases the occurrence of warfarin-associated ICH by more than 80-fold [37]. Recently, a number of new orally active anticoagulants have become available: dabigatran, a direct thrombin inhibitor, and rivaroxaban and apixaban, Factor Xa antagonists. Initial clinical trials suggest that these may induce less ICH than warfarin [38].

#### Hemorrhagic transformation

It should be noted that some patients with ischemic stroke will undergo hemorrhagic transformation, i.e. hemorrhaging within the ischemic area [3,39]. Such hemorrhagic transformation is a particular concern with tissue plasminogen activator (tPA)- induced reperfusion therapy for ischemic stroke. It led to a number of failed clinical trials of thrombolytics, with the adverse effect of increased hemorrhage offsetting the benefit of reperfusion, before the pivotal NINDS clinical trial [40] demonstrated a benefit for tPA. Such hemorrhagic transformation is not the focus of this review on primary spontaneous ICH. The reader is referred to several excellent reviews on vascular dysfunction in hemorrhagic transformation [3,39,41].

### Mechanisms underlying hematoma expansion

That some patients continue to bleed (hematoma expansion) after the initial ictus, suggests insufficient coagulation. There have, therefore, been attempts to prevent hematoma expansion by manipulating the coagulation cascade. Notably, there was the trial of factor VIIa [42]. That trial found a reduction in hematoma expansion but there was no improvement in outcome. This may have been related to adverse side effects (particularly systemic) of factor VIIa and the fact that only the 30% of patients that undergo hematoma expansion would be expected to benefit. There has, therefore, been interest in identifying which patients are liable to undergo hematoma expansion (e.g. those with the ‘spot sign’ on computerized tomography angiography; [13]) and show most benefit from treatment.

Other techniques that might limit hematoma expansion have been examined. Recently, there has been considerable interest on whether reducing blood pressure will limit hematoma expansion (the INTERACT, ATACH and ICH ADAPT clinical trials, [4,5]). The results of the phase 3 INTERACT2 trial were reported last year [43]. It showed the apparent safety of acutely lowering blood pressure in ICH patients and suggested a potential benefit on clinical outcome, although this did not quite reach statistical significance. Although the underlying rationale for this (and other) blood pressure lowering trials was to lower hematoma expansion, this phase 3 trial, in contrast to the prior phase 2 trial, failed to show a significant effect of blood pressure reduction on such expansion [43,44]. Even earlier lowering of blood pressure may have a more beneficial effect.

### Mechanisms underlying secondary BBB dysfunction in ICH

The BBB is formed by the cerebral endothelial cells and their linking tight junctions. The tightness of those junctions and a relatively low level of transcytosis results in the BBB having a very low permeability for many compounds unless they are lipophilic or are subject to carrier-mediated transport. In addition, under normal conditions, there is a very low rate of leukocyte migration into brain across the cerebral endothelium. Perivascular cells (e.g. pericytes and astrocytes) and endothelial-extracellular matrix interactions also have a role in regulating BBB function, forming the so called neurovascular unit [45-49]. In addition, leukocytes can also modulate BBB function upon adhesion and during and after transmigration across the endothelium [50].

An increase in BBB permeability and leukocyte infiltration into brain occurs in many neurological conditions. Such an increase may occur via changes in the paracellular (alterations in tight junction function) and/or the transcellular route (transcytosis) across the cerebral endothelium [51]. However, while changes in the para- and transcellular pathways across the endothelium are the ultimate cause of increased permeability after ICH, the effects of ICH may occur either directly on the endothelium or indirectly via effects on other cell types or extracellular matrix that regulate or affect the endothelium. The purpose of this section is to outline what ICH-factors may be involved in inducing BBB dysfunction (hyperpermeability and leukocyte transmigration), the mechanisms involved and the potential impact on ICH-induced brain injury.

**Figure 4:**
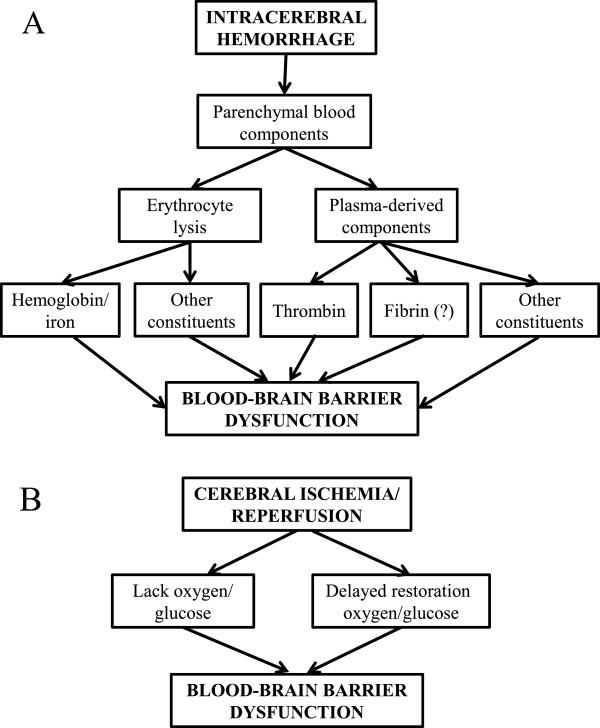
**The underlying cause of secondary BBB dysfunction appears to differ in ICH (A) from cerebral ischemia (B).** In ICH, evidence indicates a major role for the presence of blood components in brain parenchyma activating a number of pathways (cell injury, receptor-mediated signaling and inflammation) leading to BBB dysfunction. In contrast, in cerebral ischemia, the initiating cause of injury is the lack of oxygen and glucose supply to the brain. Delayed restoration of blood flow can also induce BBB dysfunction (reperfusion injury). With ischemic injury, there are a number of factors that can enhance the BBB dysfunction. Thus, for example, hyperglycemia and tissue plasminogen activator (tPA) both can result in hemorrhage after reperfusion. Less is known about factors that enhance secondary BBB dysfunction in ICH.

ICH induces delayed BBB hyperpermeability [9-11]. A potential cause could be ICH-induced ischemia, with the hematoma mass increasing intracranial pressure and reducing cerebral blood flow (a mass effect), as cerebral ischemia also increases BBB permeability [48]. However, the role of ischemia in ICH-induced brain injury is controversial [5,7]. Measurements in animal ICH models and a number of human studies indicate that perihematomal blood flows are not markedly reduced and do not reach the levels required to induce ischemic brain damage [52-54]. Indeed, reductions in blood flow may be a result rather than the cause of brain damage (reduced tissue oxygen demand and brain edema may reduce blood flow; [52,54]). The apparent absence of ICH-induced ischemia suggests that there are either components in blood that increase permeability directly or that the response to those components (e.g. inflammation) causes the hyperpermeability, and that the initiating causes of BBB dysfunction differ from cerebral ischemia (Figure 4). It should be noted, though, that there may be similarities in some of the downstream pathways activated to enhance BBB permeability (e.g. inflammation and tight junction rearrangement).

### Blood components and secondary blood–brain barrier dysfunction in ICH

#### Thrombin

Thrombin is generated from plasma prothrombin after an ICH for hemostasis. Intracerebral injection of thrombin causes BBB disruption [55,56]. Many actions of thrombin are mediated by protease activated receptors (PARs) and particularly PAR-1 [57]. Evidence indicates that BBB disruption by thrombin is at least partly PAR mediated [56,58] and that thrombin phosphorylates Src kinases to induce endothelial disruption [56]. A PAR-1 antagonist blocked the BBB disruption induced by intraventricular hemorrhage [59]. It should be noted, however, that intracerebral thrombin induces an influx of leukocytes into brain [60] and activates microglia [61] and, as discussed below, inflammation may also cause BBB disruption. Thus, thrombin may have both direct and indirect effects on BBB function.

#### Fibrin

There may be other components in or derived from plasma involved in BBB disruption and brain injury after ICH. For example, after bleeding or BBB disruption, fibrinogen enters the brain and it is cleaved to form fibrin. In multiple sclerosis, there is evidence that extravascular fibrin is important in neuroinflammation and particularly microglia activation [62,63]. Although the role of fibrin/fibrinogen in ICH-induced BBB disruption and brain injury has not been studied, microglia activation has been implicated in both (see below). The role of fibrin/fibrinogen and other plasma proteins in ICH deserves further attention.

#### Erythrocyte components

Erythrocytes are another major component of intracerebral hematomas and the role of erythrocytes and erythrocyte components in ICH-induced injury has received much attention [5,7,64]. Erythrocytes within the intracerebral hematoma eventually lyse with hemoglobin release and that hemoglobin can undergo degradation leading to iron release [5,7,65] and intracerebral injection of lysed erythrocytes, hemoglobin and iron all cause BBB disruption [66-69].

Although there may be other erythrocyte components involved in ICH-induced brain injury [70], these results suggest that the release and degradation of hemoglobin to iron after erythrocyte lysis contributes to ICH-induced BBB hyperpermeability. This hypothesis is supported by data indicating reduced ICH-induced edema with administration of an iron chelator, deferoxamine [71-73], or a heme oxygenase (HO) inhibitor, zinc protoporphyrin [74,75]. Heme oxygenases breakdown heme into biliverdin, carbon monoxide, and iron [64]. Two isoforms of HO are present in brain, the constitutive HO-2, and the inducible HO-1 (including very marked induction by ICH [76,77]). Interestingly, the effects of gene knockout (KO) on ICH-induced brain injury differ for the two isoforms, the HO-1 KO being protective while injury is greater in the HO-2 KO, which may reflect the different cellular distribution of these isoforms [78,79]. It should also be noted, though, that hemoglobin itself, as well as its degradation products may be involved in inducing BBB dysfunction [68]. There is also evidence that unconjugated bilirubin, a metabolite of biliverdin, can also induce brain edema and inflammation which may be linked to BBB effects [80].

It appears, therefore, that multiple factors produced after erythrocyte lysis can affect BBB function, although iron has a prominent role. Iron is generally thought to cause BBB dysfunction by promoting the generation of free radicals [81,82]. Oxygen and nitrogen free radicals (e.g. superoxide, hydroxyl and peroxynitrite radicals) can cause endothelial cell damage but also activate cell signaling pathways that regulate BBB permeability [83,84]. Therapeutically, an iron chelator, deferoxamine, is now in phase II clinical trial for ICH [85].

### Inflammation and secondary BBB dysfunction in ICH

Blood components also induce an inflammatory response in the perihematomal area. This includes perihematomal leukocyte infiltration and microglia activation, as well as elevations in cytokines and chemokines and the production and activation of matrix metalloproteinases (MMPs) [7,86-88]. There is evidence that each of these elements of the inflammatory response can alter BBB function from studies in ICH (see below) and other neurological conditions [50]. In addition, changes in the BBB modulate the inflammatory response. For example, the expression of adhesion molecules on the cerebral endothelium is integral in leukocyte transmigration [50,89] and infiltrating leukocytes are a major source of cytokines and MMPs after ICH [90,91].

#### Neutrophils

In rodent models of ICH, neutrophils are the first leukocytes to invade perihematomal brain tissue, with entry as early as 4 hours and a peak at 48–72 hours [88,92,93]. Neutrophils are also found around hemorrhage and in CSF after ICH in patients (reviewed in [88]). In animal models, depleting neutrophils reduces ICH-induced brain injury including secondary BBB disruption [90,94]. Similarly, toll-like receptor 4 is important in neutrophil entry into brain after ICH and toll-like receptor 4 knockout mice have reduced neutrophil extravasation and reduced ICH-induced brain injury [95]. The toll-like receptor 4-knockout mouse also has reduced macrophage infiltration and microglia activation [96,97]. Neutrophils are very important sources of TNFα [91] and matrix metalloproteinase-9 (MMP-9) [90] after ICH and may, thereby, influence the entry of other leukocytes into brain and BBB disruption.

#### Monocytes

**Figure 5:**
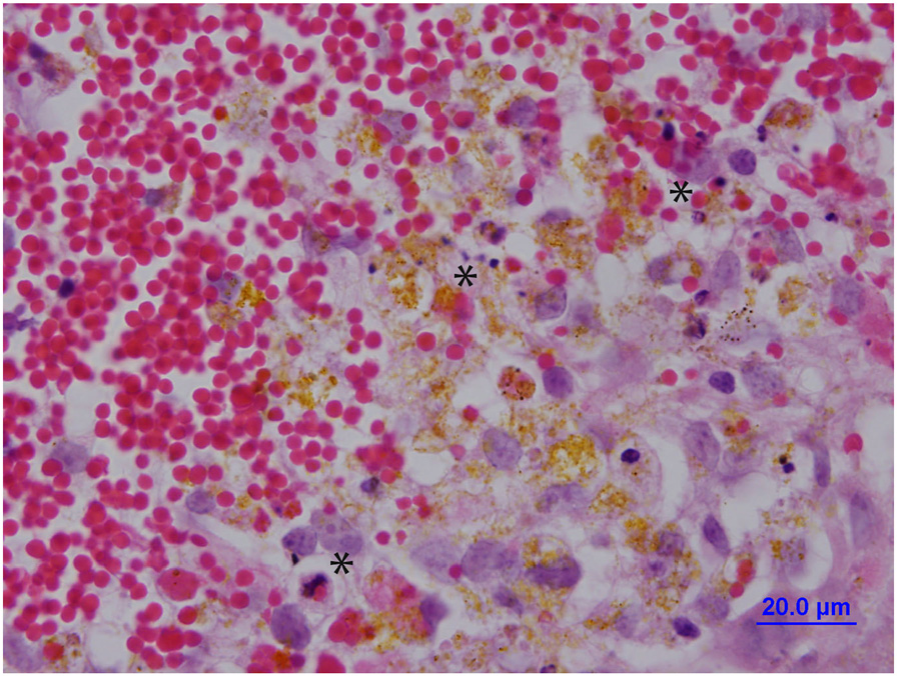
**During hematoma resolution after ICH, there is phagocytosis of erythrocytes by invading macrophages/microglia.** Hematoxylin & eosin stained section from a pig ICH model (described in [10]) at day 7 after blood injection. Note the phagocytes engulfing erythrocytes (*) and the production of hemosiderin, a breakdown product of hemoglobin (yellow staining).

Monocytes also invade perihematomal brain tissue. Thus, Hammond *et al*. [98] found marked invasion of blood-derived monocytes at day 3 after ICH in the mouse, with elevated levels still found at day 7. Two populations of monocytes were detected, CCR2^+^Ly6C^hi^ and CX3CR1^+^Ly6C^−^. The former are inflammatory and they peaked in brain before the latter which may have a role in healing [98]. Depletion of inflammatory monocytes reduced early ICH-induced brain injury. Injury was also reduced in Ccr2-knockout mice and chimeric mice with Ccr2^−/−^ hematopoietic cells [98]. While monocytes may have an adverse effect early in injury, they may also have a role in tissue repair and hematoma phagocytosis later after ICH [86,98,99] (see below; Figure 5).

#### Lymphocytes

The role of lymphocytes in ICH has received relatively little attention. Xue and Del Bigio [93] noted an influx of CD8a-immunoreactive lymphocytes into the brain between two and seven days after ICH. Peeling *et al*. [100] found that the immunosuppressant FK-506 (tacrolimus) reduced lymphocyte extravasation after ICH. Rolland *et al*. [101] found a similar effect with fingolimod, a sphingosine 1-phosphate receptor analog. Both FK-506 and fingolimod also improved functional outcome, although it should be noted that the effects of both drugs were not limited to reducing lymphocyte infiltration into brain. Whether lymphocytes play a role in BBB dysfunction after ICH has not been examined.

In cerebral ischemia and other neurological conditions such as multiple sclerosis, there has been in depth investigation of the mechanisms involved in leukocyte transmigration into brain [50,102,103]. Transmigration is a multiple step process in which adhesion molecules on both cerebral endothelial cells and leukocytes play a central role [50,102,103]. The mechanism by which neutrophils, macrophages and lymphocytes enter the brain after ICH has received much less attention. Several studies have shown an upregulation of intercellular adhesion molecule-1 (ICAM-1) after experimental ICH [92,101,104] and Rolland *et al*. found that fingolimod blocked both ICAM-1 upregulation and lymphocyte transmigration into brain [101]. Loftspring *et al*. [80] found that unconjugated bilirubin could also induce ICAM-1 upregulation, suggesting a role of this hemoglobin breakdown product in inflammation after ICH. Ma *et al*. found upregulation of another adhesion molecule, vascular adhesion protein-1 (VAP-1) after experimental ICH [105] and VAP-1 inhibition had an anti-inflammatory effect [105]. It is likely that multiple adhesion molecules are involved in leukocyte infiltration into brain after ICH.

#### Microglia

Microglia are rapidly activated after ICH. For example, after intracerebral blood injection in the rat, activated microglia were noted by 4 hours and that activation lasted for at least 4 weeks [93]. A number of studies have shown that acute inhibition of microglia reduces ICH-induced brain injury [87,88] including BBB disruption [91,106]. It should be noted, however, that in ICH (as in other neurological conditions), microglia can have beneficial as well as detrimental effects [87,88], effects which are time-dependent [87]. Thus, microglia are involved in hematoma phagocytosis, tissue repair and can have anti- as well as proinflammatory effects [87,88]. An alternate therapeutic approach is to modify microglial activation to promote the beneficial effects. Peroxisome proliferator activated receptor (PPAR)-gamma agonists promote the M2 (phagocytic) phenotype in microglia rather than the proinflammatory M1 phenotype and PPAR-gamma agonists have shown benefit in animal ICH models [86,99]. Pioglitazone, a PPAR-gamma agonist, is now in clinical trial for ICH [107].

#### Cytokines

Cytokines are signaling molecules that play a central role in inflammatory processes. A variety of cytokines are overexpressed in perihematomal tissue after ICH in animals and man [108,109] and such changes are also found at the protein level. For example, perihematomal TNFα is markedly increased by 2–4 hours in rat ICH [110] and Sun *et al*. found prolonged increases in perihematomal IL-1β [111]. Some cytokines are known to increase BBB permeability by activating signaling pathways within the endothelium and by indirect effects on other cells of the neurovascular unit [48,112,113]. In ICH, King *et al*. [114] found a TNFα receptor antagonist could reduce ICH-induced BBB hyperpermeability. Other studies have shown that curcumin [115], erythropoietin [116] and a combination of dexamethasone and melatonin [117] decrease cytokine levels and reduce BBB permeability after ICH.

Chemokines regulate chemotaxis, including the migration of leukocytes. Genomic studies have shown that a range of chemokines are overexpressed in perihematomal tissue after in animal and human ICH [108,109] and this may relate to the leukocyte influx that occurs after hemorrhage. Ma *et al*. found protein levels of monocyte chemoattractant protein 1 (CCL2) were increased after experimental ICH [105]. Yao and Tsirka [118] investigated the effects of genetic deletion of CCL2 and its receptor, CCR2, on ICH-induced brain injury. They found that CCL2 or CCR2 deletion reduced hematoma size in a mouse collagenase ICH model, reduced early microglial activation/migration but increased microglia numbers later after ICH. The deletions also reduced leukocyte infiltration. Apart from regulating leukocyte migration and microglial activation, CCL2 can also increase BBB permeability by inducing the internalization of transmembrane tight junction proteins [119-122]. These results suggest that chemokines may play an important role in BBB dysfunction after ICH.

#### Matrix metalloproteinases

MMPs play a central role in neuroinflammatory processes by degrading elements of the extracellular matrix. Intracerebral injection of bacterial collagenase (a form of MMP) causes hemorrhage [123] and a number of MMPs are upregulated in animal ICH models (e.g. MMP-2, −3, −9 and −12 [124]). MMPs may come from different sources. For example, astrocytes can produce MMP-2, microglia can produce MMP-3 and −12, and neurons can produce MMP-3, but infiltrating neutrophils are a major source of MMP-9 [124], indeed the reduced brain and BBB injury found with neutrophil depletion in rat ICH may be related to reduced MMP-9 levels [90]. There is also evidence for MMP upregulation in ICH patients in blood, CSF and perihematomal tissue (reviewed in [125]). MMP-2 and −9 are upregulated in brains of patients with cerebral amyloid angiopathy including in areas far from the acute ICH [126,127]. There is, therefore, interest in the role of MMPs in inducing ICH and in secondary brain injury, including BBB disruption.

Although MMPs are best known for extracellular matrix degradation, there is evidence of MMP-mediated cleavage of the BBB tight junction proteins, occludin and claudin-5 [128-131]. Thus, MMPs may affect BBB function and hemorrhage by multiple mechanisms, including disruption of the physical support by the endothelial basement membrane, altered endothelial: extracellular matrix signaling, promotion of leukocyte migration and direct effects on tight junction proteins.

The potential role of MMPs in ICH has been examined in patients by examining whether MMP expression correlates with outcome or markers of injury and in animals by examining the effects of MMP inhibition or genetic deletion on ICH. Thus, several studies have shown that MMP-9 expression correlates with hematoma expansion, perihematomal edema and neurological deterioration in ICH patients (reviewed in [125]). Li *et al*. recently found that increased plasma MMP-3 and MMP-9 levels were associated with worse outcome in ICH patients but not in brain edema [132]. Animal MMP inhibition/genetic deletion studies in ICH have given some conflicting results. Thus, the broad-based MMP inhibitors GM6001 [133,134] and BB-1101 [135], and genetic deletion of MMP-12 [136] reduced ICH-induced brain injury. In contrast, another MMP inhibitor, BB-94, increased ICH-induced injury [137], as did genetic deletion of MMP-9 [138]. These divergent effects of MMP manipulation may reflect the diverse roles of different MMPs, roles which may vary with time.

### Clinical trials

There are currently a number of clinical trials for ICH therapy (Table 2). As blood components may have deleterious effects on the brain (neurons, glia as well as the BBB), and because the presence of the hematoma may cause physical disruption and increase intracranial pressure, there have been many trials of clot evacuation in ICH (see [139] for a meta-analysis). As yet, these trials have not shown a definitive benefit for surgical removal, including the recent STICH2 trial [140]. This might reflect some of the adverse side effects of surgery. There are currently a number of clinical trials using minimally invasive surgery in combination with clot lysis. An example is the current phase III Minimally Invasive Surgery plus rtPA for Intracerebral Hemorrhage Evacuation (MISTIE) trial which uses t-PA to aid in clot removal [141]. This has recently been reported to reduce perihematomal edema which may suggest reduced BBB permeability [142].

**Table 2:** **Current and recently (2010 and later) finished clinical trials for treating ICH**^**#**^

**Intervention**	**Study name**	**Clinical trial #**	**Target**	**Outcome**
Surgical Evacuation	STICH II		Multiple	No benefit [140]
Surgical Evacuation/tPA	MISTIE III	NCT01827046	Multiple	Ongoing
Blood pressure lowering agents	ATACH-II	NCT01176565	Hematoma expansion	Ongoing
Blood pressure lowering agents	INTERACT2	NCT00716079	Hematoma expansion	Inconclusive*[43]
Blood pressure lowering agents	ICH-ADAPT	NCT00963976	Hematoma expansion	Ongoing
Factor VIIa (for spot sign patients)	SPOTLIGHT	NCT01359202	Hematoma expansion	Ongoing
Factor VIIa (for spot sign patients)	STOP IT	NCT00810888	Hematoma expansion	Ongoing
Factor VIIa (reversal anticoagulants)		NCT00770718	Hematoma expansion	Terminated (poor recruitment)
Platelet Transfusion		NCT00699621	Hematoma expansion	Ongoing
Pioglitazone	SHRINC	NCT00827892	Hematoma resolution	Ongoing
Deferoxamine	iDEF	NCT02175225	Iron toxicity	Ongoing
Minocycline	MACH	NCT01805895	Inflammation	Ongoing
Simvastatin		NCT00718328	Multiple	Terminated (poor recruitment)
Fluoxetine		NCT01737541	Motor recovery	Ongoing
Albumin		NCT00990509	Multiple	Terminated (PI move)
Hypothermia		NCT01866384	Multiple	Ongoing
Ibuprofen		NCT01530880	Fever prevention	Ongoing

Details on clinical trials can be found from the NIH (http://www.clinicaltrials.gov) and The Internet Stroke Center (http://www.strokecenter.org/trials/).

^#^For older clinical trials, the reader is referred to Keep *et al*. [5].

*The effect of acute blood pressure lowering did not quite reach statistical significance in the phase 3 INTERACT2 trial.

A potential concern of such trials is that surgery or surgery plus tPA might cause the release of factors from the hematoma (e.g. hemoglobin and iron) that are detrimental to the brain parenchyma and the brain vasculature. It is possible that the iron chelator, deferoxamine, which is now in phase II clinical trial for ICH [85], might be of benefit as an adjunct therapy for such clot removal approaches.

As noted above, there are a number of clinical trials focused on acutely reducing blood pressure to limit hematoma expansion [4]. Another pharmacological approach to affect hematoma size is to promote clot resolution. Microglia/macrophages are involved in clot phagocytosis and this is stimulated by PPAR-gamma agonists, such as pioglitazone [86,99]. It should be noted that PPAR-gamma agonists may have other beneficial effects (anti-inflammatory and anti-oxidant) in ICH. There is a current trial (SHRINC) of pioglitazone in ICH [107].

## Conclusion

Intracerebral hemorrhage has a variety of etiologies and the mechanisms causing the initial hemorrhage and subsequent BBB disruption may differ with etiology. Therapeutic approaches may also need to differ (e.g. between hypertensive ICH and that following amyloid angiopathy). However, since our last short review on BBB function in ICH [143], there has been progress made in understanding the mechanisms that underlie initial hemorrhage and secondary BBB dysfunction at the molecular, cellular and tissue level. There are a number of promising current clinical trials for ICH [4] that may limit those processes. Hopefully, the first successful treatments for this devastating form of stroke are not too long in the future.

## Abbreviations

AVMs: Ateriovenous malformations; BBB: Blood–brain barrier; CAA: Cerebral amyloid angiopathy; CBP: Collagen binding protein; CCMs: Cerebral cavernous malformations; HO: Heme oxygenase; ICAM-1: Intercellular adhesion molecule-1; ICH: Intracerebral hemorrhage; MMP: Matrix metalloproteinase; PAR: Protease activated receptor; PPAR: Peroxisome proliferator activated receptor; SAH: Subarachnoid hemorrhage; tPA: Tissue plasminogen activator; VAP-1: Vascular adhesion protein-1.

## Competing interests

The authors declare that they have no competing interests.

## Authors’ contributions

RFK drafted and wrote the manuscipt. NZ, JX, AVA, YH and GX commented on and revised the manuscript. All authors have read and approved the final version of the manuscript.
